# 24 Month Longitudinal Data in Ambulant Boys with Duchenne Muscular Dystrophy

**DOI:** 10.1371/journal.pone.0052512

**Published:** 2013-01-11

**Authors:** Elena Stacy Mazzone, Marika Pane, Maria Pia Sormani, Roberta Scalise, Angela Berardinelli, Sonia Messina, Yvan Torrente, Adele D’Amico, Luca Doglio, Emanuela Viggiano, Paola D’Ambrosio, Filippo Cavallaro, Silvia Frosini, Luca Bello, Serena Bonfiglio, Roberto De Sanctis, Enrica Rolle, Flaviana Bianco, Francesca Magri, Francesca Rossi, Gessica Vasco, GianLuca Vita, Maria Chiara Motta, Maria Alice Donati, Michele Sacchini, Tiziana Mongini, Antonella Pini, Roberta Battini, Elena Pegoraro, Stefano Previtali, Sara Napolitano, Claudio Bruno, Luisa Politano, Giacomo Pietro Comi, Enrico Bertini, Eugenio Mercuri

**Affiliations:** 1 Department of Paediatric Neurology, Catholic University, Rome, Italy; 2 Biostatistics Unit, Department of Health Sciences, University of Genoa, Genoa, Italy; 3 Child Neurology and Psychiatry Unit, “C. Mondino” Foundation, Pavia, Italy; 4 Department of Neurosciences, Psychiatry and Anaesthesiology, University of Messina, Messina, Italy; 5 Dino Ferrari Centre, Neuroscience Section, Department of Pathophysiology and Transplantation (DEPT), University of Milan, Neurology Unit, Ca’ Granda Ospedale Maggiore Policlinico, Milan, Italy; 6 Department of Laboratory Medicine, Unit of Molecular Medicine, Bambino Gesù Hospital, Rome, Italy; 7 Neuromuscular Disease Unit, G. Gaslini Institute, Genoa, Italy; 8 Dipartimento di Medicina Sperimentale, Seconda Università di Napoli, Naples Italy; 9 Department of Developmental Neuroscience, Stella Maris Institute, University of Pisa, Pisa, Italy; 10 Department of Neurosciences, University of Padua, Padua, Italy; 11 Child Neurology and Psychiatry Unit, Maggiore Hospital, Bologna, Italy; 12 Neuromuscular Center, SG. Battista Hospital, University of Turin, Turin, Italy; 13 Metabolic and Neuromuscular Unit, Meyer Hospital, Florence, Italy; 14 Department of Neurology, San Raffaele Scientific Institute, Milan, Italy; Johns Hopkins University School of Medicine, United States of America

## Abstract

**Objectives:**

The aim of the study was i) to assess the spectrum of changes over 24 months in ambulant boys affected by Duchenne muscular dystrophy, ii) to establish the difference between the first and the second year results and iii) to identify possible early markers of loss of ambulation.

**Methods:**

One hundred and thirteen patients (age range 4.1–17, mean 8.2) fulfilled the inclusion criteria, 67 of the 113 were on daily and 40 on intermittent steroids, while 6 were not on steroids. All were assessed using the 6 Minute Walk Test (6MWT), the North Star Ambulatory Assessment (NSAA) and timed test.

**Results:**

On the 6MWT there was an average overall decline of −22.7 (SD 81.0) in the first year and of −64.7 (SD 123.1) in the second year. On the NSAA the average overall decline was of −1.86 (SD 4.21) in the first year and of −2.98 (SD 5.19) in the second year. Fourteen children lost ambulation, one in the first year and the other 13 in the second year of the study. A distance of at least 330 meters on the 6MWT, or a NSAA score of 18 at baseline reduced significantly the risk of losing ambulation within 2 years.

**Conclusions:**

These results can be of help at the time of using inclusion criteria for a study in ambulant patients in order to minimize the risk of patients who may lose ambulation within the time of the trial.

## Introduction

In the last few years the increasing number of potentially effective therapeutical approaches for patients affected by Duchenne muscular dystrophy (DMD) has stimulated the interest in outcome measures to be used in clinical trials. The 6 minute walk test (6MWT) has been recently chosen as the primary outcome measure as it provides a global assessment of functional mobility, endurance, and ability to walk. Its use has been reported in international multicenter clinical trials and longitudinal natural history studies in DMD ambulant patients [1], [2], [3], [4]. Functional scales, such as the North Star Ambulatory Assessment (NSAA) represent an ideal additional tool to the 6MWT, as they provide information on a wider spectrum of functions that reflect everyday life activities [5]. The NSAA has been specifically developed for ambulant DMD boys and has also been validated and used in multicentric longitudinal studies [3], [4], [6], [7], [8].

We have recently reported data from a multicentric longitudinal study, describing the changes on both 6MWT and NSAA observed over 12 months in ambulant DMD boys.

In the present study we report the results at 24 months using the same assessments. The aim of the study was to assess the spectrum of changes over 24 months in the individual measures and their correlation with age and possible treatment with steroids. We were also interested to establish the difference between the first and the second year results in the cohort and whether, given a longer follow up, we could identify early markers of events such as loss of ambulation.

## Subjects and Methods

The study is a longitudinal multicentric cohort study involving 11 tertiary neuromuscular centers in Italy. Patients were recruited between January 2008 and June 2009 and followed for at least two years with the last follow up visit performed in July 2011. The study was approved by the Ethical Committee of each centre.

Patient inclusion criteria at baseline were: genetically proven DMD diagnosis, patient still ambulant and able to walk independently for at least 75 meters, no severe or moderate learning difficulties or behavioral problems. Genetic and treatment information were collected and classified following the criteria used in our previous study [4]. All patients attending the 11 participating centers who fulfilled the inclusion criteria were enrolled in the study. As part of the routine assessments in all centers patients are seen at least once every 12 months and all centers performed at each visit the NSAA followed by the 6MWT. Data were collected from the first assessment after recruitment (baseline) from the 12 month and the 24 month follow up assessment.

As the various centers used different types of steroids (deflazacort and prednisone) and had different regimes, we broadly subdivided our cohort into: a) no steroids: this included boys who had never been on steroids and others who had used them for less than a year and had stopped treatment at least one year before the study; b) intermittent regime, patients who had been, at the time of the study, on alternate days or alternate weeks or 10 days on/10 days off of either .75 mg of prednisone or .9 mg/kg/day of deflazacort for at least a year; c) daily regime, patients who had been, at the time of the study, on daily treatment of .75 mg of prednisone or .9 mg/kg/day of deflazacort for over a year, also including those in whom the dose had not been always completely adjusted to the current weight. A small number of patients who took deflazacort on alternate days but with a dose of approximately 2 mg/kg were also included in this group as their monthly dose was similar if not higher to those with a standard daily dose of steroids.

Details of the training for the physiotherapists involved in the study and of the interobserver reliability for NSAA among the centers have already been reported [3], [4], [6].

### Standard Protocol Approvals, Registrations, and Patient Consents

As this was an observational study, requiring non invasive procedures of assessment that were used routinely in all centers and did not require extra visits to the centres, no informed consent was required as the data were analyzed anonymously. Parents of participants (all our patients were minor/children) and patients were informed that the data collected as part of our routine clinical assessment were going to be used anonymously for an observational study defining natural history of the diseases and they all gave verbal consent. Written consent was not required as the data were analyzed anonymously. The Ethical committee from Catholic University, Rome approved this procedure. All clinical investigation was conducted according to the principles expressed in the Declaration of Helsinki.

### 6MWT

6MWT was performed in all DMD ambulant boys older than 5 according to the ATS guidelines [9], modified by having two examiners, one recording time and distances and one staying close to the patient for safety issues.

### NSAA

The scale consists of 17 items, ranging from standing (item 1) to running (item 17) and includes several items assessing abilities that are necessary to remain functionally ambulant, items assessing abilities, such as head raise and standing on heels that can be partly present in the early stages of the disease and a number of activities such as hopping, or running that are generally never fully achieved in untreated DMD boys but that have been found in those treated with daily steroids.

Each item can be scored on a 3 point scale using simple criteria: 2 -Normal achieves goal without any assistance; 1 -Modified method but achieves goal independent of physical assistance from another person; 0 - Unable to achieve independently.

A total score can be achieved by summing the scores for all the individual items. The score can range from 0, if all the activities are failed, to 34, if all the activities are achieved.

### Timed Items

The NSAA also includes the possibility to record timed items (10 meter timed walk/run test and time to rise from the floor or Gower test) [6], [8]. The time taken to complete the task is not part of the global score but provides an additional measure of the DMD boys’ abilities that can be monitored over time. The 10 meter timed test is usually expressed as the time spent for walking/running 10 meters. As in children who are or become unable to perform this task the time cannot be measured, conventionally a time equal or bigger than the worst performance in the group is subjectively given to indicate poor performance.

### Statistical Analysis

Functional scales were evaluated longitudinally over a 24 months period of time. The dependence of NSAA, 6MWT, 10 m and Gowers test on time was evaluates using a mixed random effect model, adjusting for baseline age (< = 7 vs >7 years) and the use of steroids at baseline (alternate vs continuous). The age of 7 was chosen according to our previous findings showing that 7 years appear to be the age when DMD boys have a slope of deterioration [4] and to identical results in other cohorts [5]. Correlations were evaluated by the Spermann rank correlation coefficients.

The effect of baseline assessments on the risk to become unable to walk was evaluated by a Receiver operating characteristic (ROC) analysis that indicates the cut-off value maximizing at the same time the sensitivity and the specificity of that variable.: For each baseline variable the cut-off value better indicating an increase in the risk of loss of the ambulation ability was determined.

Logistic univariate analysis were used to estimate the impact of each baseline factor on the risk of loss of ambulation.

## Results

One hundred and thirteen patients fulfilled the inclusion criteria and entered the study.

Ninety-six of these patients have already been reported in our previous study reporting 12 month follow up in 106 ambulant DMD boys [4]. Six of the 106 were lost at follow up, three entered a stem cell study and one died. Seventeen new patients who were recruited using the same protocol and had not completed the 12 month assessment at the time of the first study were added to the present study. All the tests were performed safely without any mayor fall during the performance of the individual measures. Of the 113 boys, 67 were on daily (mean age 8,5) and 40 on intermittent steroids (mean age 7,8), and 6 were not on steroids (mean age 6,9).

### 6MWT

Three children lost the ability to perform the test within 1 year and another 13 within 2 years from baseline (table 1). The 6MWD showed an average overall decline of −22.7 (SD 81.0) meters in the first year and of −64.7 (SD 123.1) in the second year.

**Table 1:** Details of number of patients who lost the ability to perform the individual tests at baseline, 12 and 24 months.

	BASELINE	1 YEAR	2 YEARS
GOWERS TEST	8	16	34
6MWT	0	3	16
10 m	0	1	13
NSAA	0	1	9
Loss of ambulation	0	1	13

**Figure 1:**
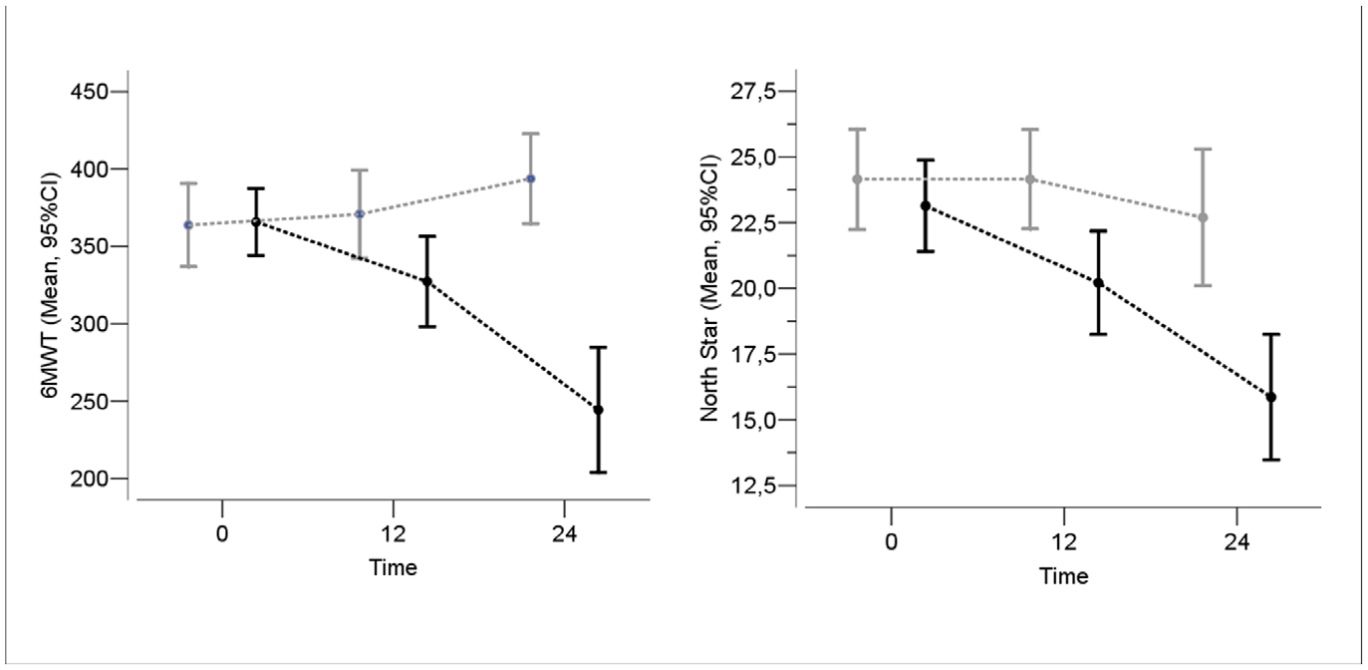
Mean changes in 6MWT and in NSAA scores in patients below the age of 7 years at baseline (grey line) and above (black line).

The changes were significantly different in the two baseline age groups (test for interaction, p<0.001): children below 7 remained on the average stable with also a slight increase during the first year (mean 6MWT change = +18.7 (SD = 71.4)) and also during the second year (mean 6MWT change = +14.6 (SD = 67.3)). Children above 7 had a decrease of about 42 meters during the first year (mean 6MWT change = −41.8, SD = 78.3) and about 80 meters during the second year (mean 6MWT change = −80.6, SD = 96.7) (Figure 1).

The changes were not significantly different according to steroid treatment (alternate vs continuous) (p for interaction = ns).

### NSAA

One child lost the ability to perform the test within 1 year and another 8 within 2 years from baseline (table 1). There was an average overall decline of −1.86 (SD 4.21) in the first year and of −2.98 (SD 5.19) in the second year.

The changes were significantly different in the two baseline age groups (test for interaction, p<0.001): children below 7 remained on the average stable during the first year (mean NSAA change = +0.15 (SD = 4.8)) and started to decrease during the second year (mean NSAA change = −0.78 (SD = 5.1)). Children above 7 had a decrease of about 3 points during the first year (mean NSAA change = −3.0, SD = 3.3) and higher than 4 points during the second year (mean NSAA change = −4.2, SD = 4.8) (Figure 1).

### Timed Items

Eight children were unable to perform the Gower test at baseline. Other 8 lost the ability to perform it within 1 year and and another 18 within 2 years. The changes in the time taken to perform the manoeuvre in the subjects who were still able to perform it showed an average overall decline of 5.05 seconds (SD 12.45) in the first year and of 7.83 (SD 16.01) in the second year.

The changes were significantly different in the two baseline age groups (test for interaction, p = 0.003): children below 7 had a mean decrease of 1.75 seconds during the first year (SD = 4.62) and of 1.79 sec during the second year (SD = 7.33). Children above 7 had a decrease of 6.88 seconds during the first year (SD = 14.87) and of 11.32 during the second years (SD = 18.59).

During the first year only 1 child lost the ability to walk (i.e not being able to take more than 5 steps) and to perform all tests. In the second year another 12 children lost ambulation. Nine where unable to perform any test while other 5 were unable to perform 6MWT and timed tests but were still able to perform some items on the NSAA (Table 1).

### Factors Predicting the Probability of Loss of Ambulation after 2 Years

**Figure 2:**
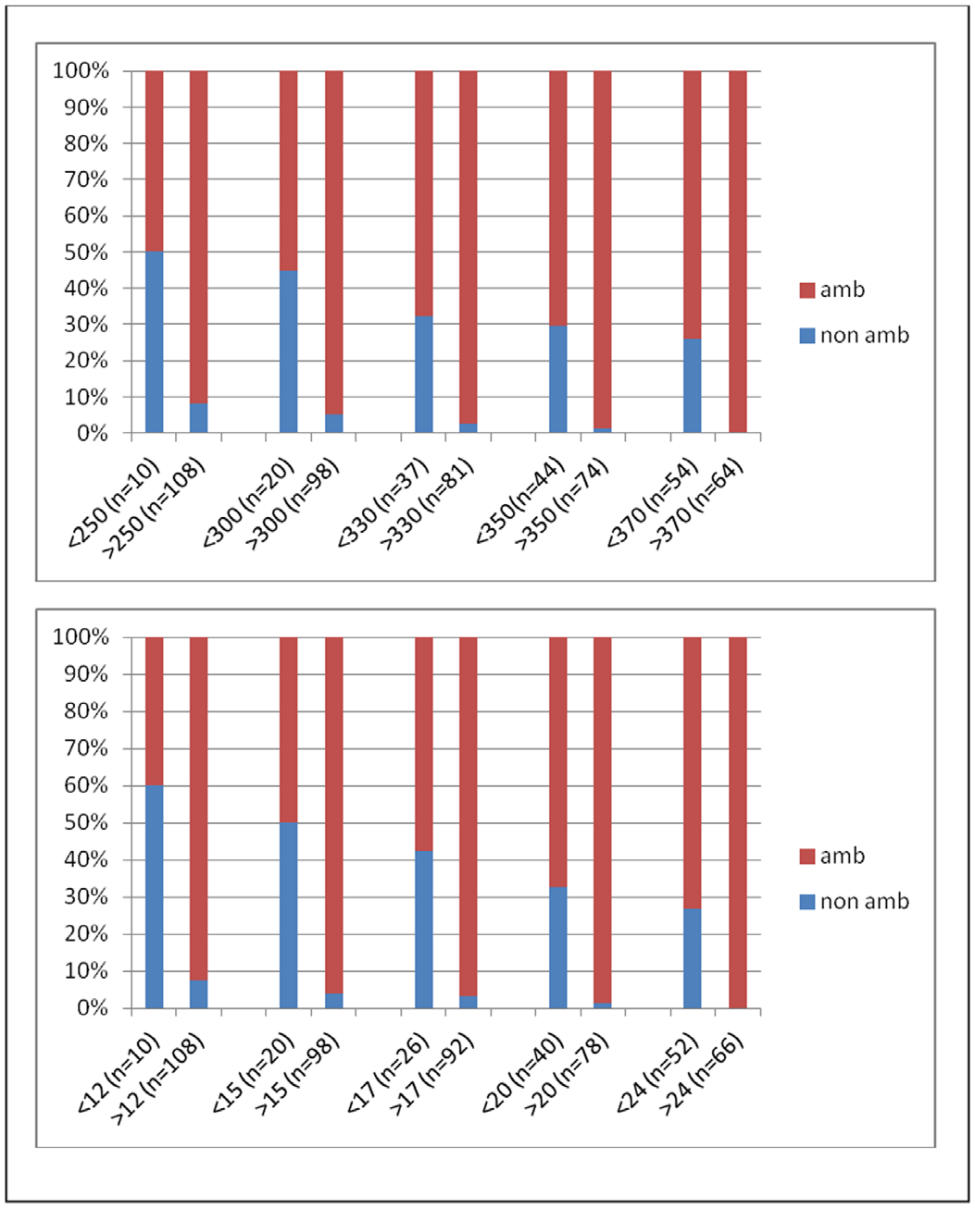
Risk of losing ambulation within 2 years in relation to different cut-off points of 6MWD and NSAA scores at baseline.

Cut-off values for each baseline variable giving the maximum sensitivity and specificity were estimated from the ROC curves: the NSAA value best discriminating patients at high risk of ambulation loss was 22, the best 6MWD cut-off was 330 m, the best 10 m cut-off was 7 sec and the best cut-off for the Gowers assessment was 7.2 sec. In Table 2 the association between baseline variables value (above and below the optimized cut-off values) are reported. All the children who lost ambulation were older than 7. Figure 2 shows the risk of losing ambulation within 2 years using different cut off points for 6MWT and NSAA.

**Table 2:** Association between baseline variables value (above and below the optimized cut-off values).

	OR	95.0% C.I.		P value
		Lower	Upper	
NSAA baseline				
< = 22	ref			
>22	37.5	4.7	300.4	0.001
6MWT baseline				
< = 330	ref			
>330	23.6	4.9	113.8	<0.001
Gowers baseline				
>7.2	ref			
< = 7.2	6.2	1.6	23.6	0.007
10 m baseline				
>7	ref			
< = 7	7.9	2.2	28.3	0.002

All the children who lost ambulation were older than 7.

## Discussion

The aim of the study was to assess the spectrum of changes over 24 months using a combined approach including a measure of endurance (6MWT), a functional scale (NSAA) and timed tests in a large cohort of 113 ambulant DMD boys of age ranging between 4.1 and 17 years at baseline. All tests were well tolerated and easily administered and were safely completed.

Our results showed that the changes in 6MWD were of −87.3 m over 24 months, with a much higher change in the second year compared to the first one. There was a substantial heterogeneity with a SD of 123.1. Similar results were obtained in the NSAA with a mean change of approximately −5 points over 24 months, and a SD of 5.19.

As previously reported for the 12 month assessment [4], the changes were different according to age. Boys who were below the age of 7 at baseline had some improvement on both NSAA and 6MWT in the first year and, a small improvement on 6MWT was also found in the second year.

Those above the age of 7 in contrast showed a progressive deterioration that was much more obvious in the second year on all tests. Adding an extra year in this age group had a significantly higher impact on motor performance and increased the chances of losing ambulation.

It has recently been reported that the improved distances on the 6MWT in the younger boys should be considered the result of growth rather than a real improvement of muscle function, suggesting the use of a % predicted 6MWD calculated using an age and height-based equation that would account for growth and development [2]. Unfortunately in our study height measures were not standardized across centers as this was not planned at the time when the study started and we therefore cannot provide further evidence of the value of this analysis.

When we assessed the possible effect of steroids on changes over 24 months we could not confirm our previous findings obtained at 12 months suggesting a positive effect of daily steroids on the outcome measures [4]. On the 24 month analysis the magnitude of changes in the overall group of patients with daily regime was not significantly different than in those with intermittent regime. The similar decline between patients with daily and intermittent steroids may be explained by the fact that the study was not designed to establish the effect of steroids and the groups were not stratified according to their steroid regime or age. With few exceptions, the patients who were able to walk beyond the age of 10 and all above the age of 12 were on daily steroids and the group on daily treatment was on average one year older than those on intermittent regime and therefore at higher risk of sudden deterioration. This should therefore be further explored with prospective studies with appropriate stratification and randomization according to age and steroid regime.

In agreement with previous studies we also observed that the ability to performed Gowers test was lost before boys failed the other tests [10], with eight boys already unable to get up from the floor at baseline, at the time when they were still able to walk at least 75 meters. The number of patients failing this ability progressively increased at 12 and 24 months, followed by the inability to perform the 6MWT, and in a smaller number of patients by the 10 meters timed test. As the NSAA also includes activities such as lifting head or standing that can be retained even after loss of ambulation, there were a number of patients in whom NSAA scores could be recorded even after they were unable to perform all the other tests. Additional measures, such as those assessing upper limb activities, could have helped to follow possible changes of other functional abilities after loss of ambulation.

We were also interested to establish whether we could identify cut off points able to predict loss of ambulation or, conversely to define a cohort with a low risk of losing ambulation within two years. This information appears to be useful at the time of recruiting DMD boys in a clinical trial in which the primary outcome measures is the 6MWT that can only be performed in ambulant patients.

While only one boy lost ambulation at one year from baseline, another 12 lost the ability in the second year. The analysis of our results suggested that a 6MWD of at least 330 meters, or a NSAA score of 18 reduced significantly the risk of losing ambulation within 2 years. The same applied to walking ten meters within 7 seconds or getting up from the floor within 10 seconds. These results can be of help at the time of using inclusion criteria for a study in ambulant patients in order to minimize the risk of patients who may lose ambulation within the time of the trial.

This study provides for the first time longitudinal data using 6MWT, a functional scale and timed items over a 24 month period, providing the spectrum of changes observed in each measure and predictors of loss of ambulation. In our cohort one extra year produced a significant difference in the performance and in the standard deviations of the changes on all measures. Age and scores at baseline will help to identify the children who will lose ambulation or are more likely to show steep changes in their performance and these aspects should be considered at the time of designing a clinical trial.
