# Correction: 24 Month Longitudinal Data in Ambulant Boys with Duchenne Muscular Dystrophy

**DOI:** 10.1371/annotation/cbe611fe-cda9-4d98-9574-0ac18e109daa

**Published:** 2013-11-11

**Authors:** Elena Stacy Mazzone, Marika Pane, Maria Pia Sormani, Roberta Scalise, Angela Berardinelli, Sonia Messina, Yvan Torrente, Adele D’Amico, Luca Doglio, Emanuela Viggiano, Paola D’Ambrosio, Filippo Cavallaro, Silvia Frosini, Luca Bello, Serena Bonfiglio, Roberto De Sanctis, Enrica Rolle, Flaviana Bianco, Francesca Magri, Francesca Rossi, Gessica Vasco, GianLuca Vita, Maria Chiara Motta, Maria Alice Donati, Michele Sacchini, Tiziana Mongini, Antonella Pini, Roberta Battini, Elena Pegoraro, Stefano Previtali, Sara Napolitano, Claudio Bruno, Luisa Politano, Giacomo Pietro Comi, Enrico Bertini, Eugenio Mercuri

There was an error in the affiliation of author Antonella Pini.

The correct affiliation for this author is: Child Neurology and Psychiatry Unit, IRCCS Institute of Neurological Sciences, Bellaria Hospital, Bologna, Italy 

